# Computational methods using weighed-extreme learning machine to predict protein self-interactions with protein evolutionary information

**DOI:** 10.1186/s13321-017-0233-z

**Published:** 2017-08-18

**Authors:** Ji-Yong An, Lei Zhang, Yong Zhou, Yu-Jun Zhao, Da-Fu Wang

**Affiliations:** School of Computer Science and Technology, China University of Mining and Technology, Xuzhou, 21116 Jiangsu China

**Keywords:** SIPs, Weighed-extreme learning machine, Local average group, PCA

## Abstract

Self-interactions Proteins (SIPs) is important for their biological activity owing to the inherent interaction amongst their secondary structures or domains. However, due to the limitations of experimental Self-interactions detection, one major challenge in the study of prediction SIPs is how to exploit computational approaches for SIPs detection based on evolutionary information contained protein sequence. In the work, we presented a novel computational approach named WELM–LAG, which combined the Weighed-Extreme Learning Machine (WELM) classifier with Local Average Group (LAG) to predict SIPs based on protein sequence. The major improvement of our method lies in presenting an effective feature extraction method used to represent candidate Self-interactions proteins by exploring the evolutionary information embedded in PSI-BLAST-constructed position specific scoring matrix (PSSM); and then employing a reliable and robust WELM classifier to carry out classification. In addition, the Principal Component Analysis (PCA) approach is used to reduce the impact of noise. The WELM–LAG method gave very high average accuracies of 92.94 and 96.74% on *yeast* and *human* datasets, respectively. Meanwhile, we compared it with the state-of-the-art support vector machine (SVM) classifier and other existing methods on *human* and *yeast* datasets, respectively. Comparative results indicated that our approach is very promising and may provide a cost-effective alternative for predicting SIPs. In addition, we developed a freely available web server called WELM-LAG-SIPs to predict SIPs. The web server is available at http://219.219.62.123:8888/WELMLAG/.

## Background

All the time, protein–protein interactions (PPIs) play an important role in biological activity. However, a crucial problem regarding Self-interactions Proteins (SIPs) is whether proteins can interact with their partners. SIPs is a special type of PPIs and are those in which more than two copies of the protein can mutual effect. Two SIP partners are the same copies of the protein and can be represented by the same gene. This can lead to the formation of homo-oligomer. In recent years, many studies have found that SIPs play a key role in the evolution of protein interaction networks (PINs) and cellular functions [1]. Therefore, it is much crucial that whether a protein can self-interact for the elucidation of its functions. Knowledge of SIPs can also provide a better understanding of the regulation of protein function and disease mechanisms. Many researches have demonstrated that homo-oligomerization is an essential function for biological activity and play an important role in a wide range of biological processes, for example, signal transduction, gene expression regulation, enzyme activation and immune response [2–6]. However, owing to SIPs is a special type of protein–protein interactions, PPIs is still much important in a wide range of biological processes. Previous researchers have discovered that SIPs can variously extend the function diversity of proteins without increasing the size of genome. In addition, it is much useful for SIPs that can also strengthen the stability and prevent the denaturation of a given protein through decreasing its surface area [7]. As a result, developing a robust and effective computational approach based on protein sequence to detect SIPs has become more and more important.

Many previous studies have focused on predicting PPIs by developing computational methods. For example, Li et al. [8] proposed a novel computational approach for detecting PPIs, which uses discriminative vector machine (DVM) classifier to combine with physicochemical and evolutionary-based feature extraction methods. Jia et al. [9] developed an feature extraction method based on the physicochemical descriptors and employed the Random Forests classifier to carry out classification, which yielded good experimental results. Yang et al. [10] presented a new method based on protein sequence, which used local protein sequence descriptors as a novel representation and employed the k-nearest neighbors classifier to execute classification. Guo et al. [11] employed the SVM classifier to combine with autocorrelation feature extraction approach to identify PPIs. Ming et al. [12] used a sequence-based correlation coefficient (CC) transformation and also adopted the SVM classifier to predict PPIs. These methods usually focused on exploring the correlational information contained protein pairs, such as, coevolution, co-localization and co-expression. Nevertheless, this information is not enough for predicting SIPs. Furthermore, the datasets of prediction PPIs do not contain the PPIs between the same partners. For these reasons, these computational methods are not used to identify SIPs. In the previous study, Liu et al. [1] proposed a method for constructing a prediction model known as SLIPPER to predict SIPs, which can integrate multiple representative known properties. To the best of our knowledge, some studies about PPI have been reported very recently that may be also relevant to SIPs [13–15]. However the method has an obviously drawback since it cannot deal with the proteins not covering the current human interatomic. Because of the limitations of the aforementioned approaches, one major challenge in the study of prediction SIPs is how to exploit automated methods for SIPs detection.

In the work, we developed a novel computational approach termed WELM–LAG to predict SIPs by only using protein sequence information. The WELM–LAG method used a newly feature extraction method called Local Average Group that can capture evolutionary information from PSSM and employed an effective and robust classifier called the Weighed-Extreme Learning Machine to execute classification. The major improvement of our approach lies in adopting an effective feature extraction approach to represent candidate self-interacting proteins by exploring the evolutionary information embedded in PSI-BLAST-constructed position specific scoring matrix (PSSM); and then it also employs a reliable and robust WELM classifier to carry out classification. The proposed method was carried out on human and yeast datasets, respectively, which achieved very excellent prediction accuracies of 96.74 and 92.94%. At the same time, we also compared our method with the SVM classifier and other existing approaches on *human* and *yeast* datasets. The experimental results proved that our WELM–LAG model can extract the hidden key information beyond the sequence itself and obtain much better prediction results than previous method. It is proved that the WELM–LAG method is fit for SIPs detection and can execute incredibly well for identifying SIPs.

## Methods

### Dataset

There are 20,199 curated *human* protein sequences in the UniProt database [16]. The PPI data can be obtained from diversity resources, including DIP [17], BioGRID [18], IntAct [19], InnateDB [20] and MatrixDB [21]. In the paper, we constructed the PPIs data that only contains the same two interaction protein sequence and whose interaction type was defined as ‘direct interaction’ in relevant databases. Consequence, we acquired 2994 human Self-interactions protein sequences. For assessing the prediction performance of the proposed approach, the experiment datasets were constructed, which contains three steps [22]: (1) the protein sequences whose length less than 50 residues and longer than 5000 residues were removed from the whole human proteome;(2) in order to construct the positive dataset, we selected the SIP data that must satisfy one of the following conditions: (a) it has been detected for the Self-interactions by one small-scale experiment or at least two types of large-scale experiments; (b) Self-interactions Protein data have been defined as homooligomer (including homodimer and homodimers) in UniProt; (c) it has been reported by at least two publications for the Self-interactions;(3) to construct the negative dataset, all kinds of SIPs contained the whole human proteome (including proteins annotated as ‘direct interaction’ and more extensive ‘physical association’) and UniProt database. As a result, the resulting experiment human dataset contained 15,938 non-SIPs as negatives and 1441 SIPs as positives [22]. In addition, for further demonstrating the prediction performance of WELM-LAG, we also constructed *yeast* dataset, which contained 5511 negative and 710 positive protein sequences [22] by using the same strategy mentioned above.

### Feature extraction method

In the work, we used Position Specific Scoring Matrix (PSSM) to predict SIPs. In the experiment, each protein sequence was converted into a PSSM through employing Position Specific Iterated BLAST (PSI-BLAST) [23]. Each PSSM can be defined a $L \times 20$ matrix $P = \left\{ {P_{ij} {\text{i}}:1 = 1 \ldots L,{\text{j}} = 1 \ldots 20} \right\}$, where *P* represents the length of a given sequence, 20 are the number of 20 amino acids, and $P_{ij}$ represents the score of the $j_{th}$ amino acid in the $i_{th}$ position for a given protein sequence, where $P_{ij}$ can be expressed as $P_{ij} = \sum\nolimits_{k = 1}^{20} {m\left( {i,k} \right) \times n\left( {j,k} \right)}$, where $m\left( {i,k} \right)$ represents the appearing frequency of the $k_{th}$ amino acid at position $i$ of the probe, and $n\left( {j,k} \right)$ is the score of Dayhoff’s mutation matrix between ${\text{j}}_{\text{th}}$ and ${\text{k}}_{\text{th}}$ amino acids. Thus, a high score cab be obtained for a good conserved position, while a weakly conserved position only gets a low score.

In the study, in order to obtain highly and widely homologous protein sequences, PSI_BLAST’s e-value parameter was set to 0.001. Meanwhile, three iterations were selected. However, one major challenge in the machine learning-based methods is how to extract useful informative features. In the work, since each PSSM has different length of amino acids. As a result, each PSSM cannot be directly converted into a feature vector, which will result in different length of feature vectors. For solving this question, Local Average Group (LAG) approach is employed to create feature vectors. The Local Average Group is described as follows: a Group consists of 5% of the length of a given sequence. As a result, regardless of protein sequence’s length, we divided each PSSM of a given sequence into 20 Groups. Thus, each Group contains 20 features derived from the 20 columns of PSSMs. Related mathematical formula represented as follows:

1 $$\begin{aligned} &{\text{LAG}}\left( F \right) = \frac{20}{P}\mathop \sum \limits_{k = 1}^{{\frac{P}{20}}} Mat\left( {k + \left( {i - 1} \right) \times \frac{P}{20},j} \right) \hfill \\ &\quad i = 1, \ldots , 20;\;j = 1, \ldots , 20;\;P = j + 20 \times \left( {{\text{i}} - 1} \right), \hfill \\ \end{aligned}$$

where *P* represents the length of a given protein sequence, *P*/20 is 5% of the length of a given sequence, which represents the length of the $j_{th}$ group. The $Mat\left( {k + \left( {i - 1} \right) \times \frac{P}{20 } ,j} \right)$ represents a 1 × 20 vector captured from PSSM matrix at the $i_{th}$ position in the $j_{th}$ group. Thus, each PSSM was divided into 20 groups and expressed as a 400-dimensional feature vector. The theoretical basis of LAG is that the residue conservation tendencies are similar and the locations of domains are closely related to the length of protein sequence in the same family [24]. In our application, each protein sequence was transformed into a 400 dimensional feature vector through employing LAG method.

In the study, in order to improve prediction accuracy, the dimensionality of feature vectors was reduced from 400 to 300 through employing the PCA method. This can reduce the influence of noise. In addition, for assessing the efficient of the proposed feature extraction method, we compared it with other four methods by using the SVM classifier on yeast datasets of PPIs (the yeast dataset contains 11,188 protein pairs): Global encoding (GE) [25], auto covariance (AC) [26], auto cross covariance (ACC) [26] and local protein sequence descriptors (LD) [27]. It can be seen from Table 1, the proposed feature extraction method yielded obviously better prediction accuracy compared to other existing methods by using the same classifier.

**Table 1:** Comparison of predicting accuracy between our feature extraction method and other methods on yeast dataset

Prediction model	Ac (%)
AC + SVM	87.36
ACC + SVM	89.33
GE + SVM	91.73
LD + SVM	88.56
Our method (SVM + LAG)	93.21

**Fig. 1:**
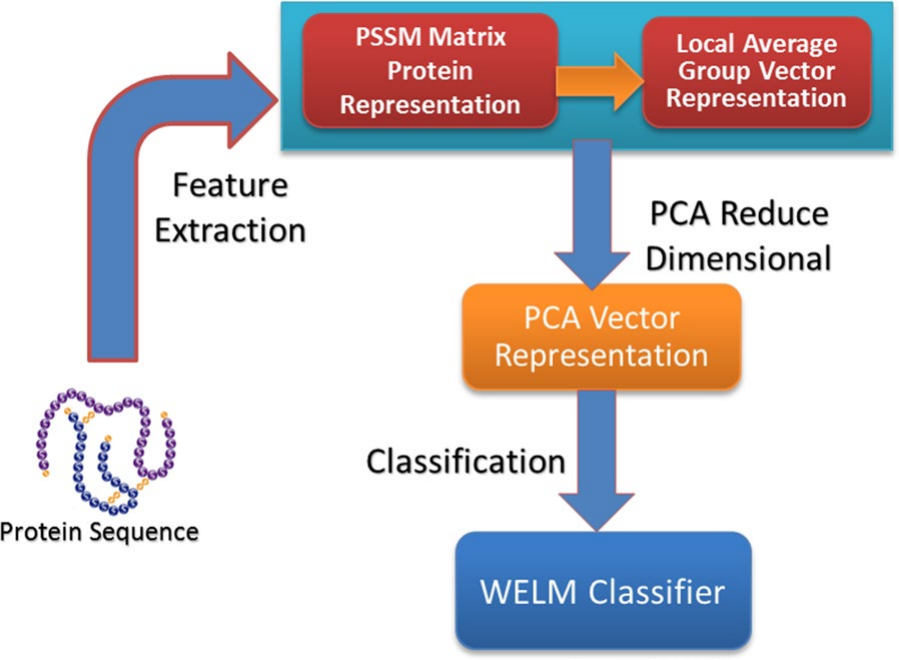
The flow chart of WELM–LAG method

In the paper, the feature extraction method based on Local Average Group combining with PCA employed to capture key feature information and the robust WELM classifier is used to execute classification. The flow of the proposed WELM–LAG method for predicting SIPs is displayed in Fig. 1.

### Weighed-extreme learning machine

The unweight Extreme learning machine can randomly generate the hidden node, which is a main characteristic that distinguishes from tradition neural network learning algorithms [28]. That is to say, it can randomly assign the parameters contained the hidden nodes for independent of the training samples. We expressed the hidden layer output as a row vector $F\left( {\text{X}} \right) = \left[ {{\text{f}}_{1} \left( m \right) \ldots f_{L} \left( m \right)} \right]$, where *m* is the input sample, *L* represents the number of hidden nodes [28]. Assume that there are training sample sets $\left\{ {m_{i} ,p_{i} } \right\}$, the model of single-hidden layer feed forward networks (SLFN) [29] can be defined as follow:

2 $$F\beta = P$$

where F represents the hidden layer output matrix, $\upbeta$ is the output weight and $P$ represents the target vector.

3 $$F = \left[ {\begin{array}{*{20}c} {\begin{array}{*{20}c} {f_{1} \left( {m_{1} } \right)} \\ {\begin{array}{*{20}c} . \\ . \\ . \\ \end{array} } \\ \end{array} } \\ {f_{L} \left( {m_{n} } \right)} \\ \end{array} } \right]$$

The minimal norm along with the least square solution can be analytically determined by employing Moore–Penrose ‘‘generalized’’ inverse $\hat{F}$

4 $${\text{when }}n < L:\upbeta = \hat{F} \div P = F^{T} \left( {\frac{I}{C} + FF^{T} } \right)^{ - 1} P$$

5 $${\text{when}}\;L < n: \beta = \hat{F} \div P = \left( {\frac{I}{C} + F^{P} F} \right)^{ - 1} F^{P} P$$

As is displayed in two formulas above that a positive value $\frac{\text{I}}{\text{C}}$ is added to the diagonal of $FF^{P}$ or $F^{P} F$ in order for better generalization performance. Users can select one of the two formulas above according to the number of training sets.

For the Weighted Extreme learning machine, for maximizing the marginal distance and minimizing the weighted cumulative error with regards to each sample, the optimization problem can be defined as follows [28]:

6 $${\text{Minimizing:}}\; \left| {\left| {F\beta - P} \right|} \right|^{2} \left| {\left| \beta \right|} \right|, \quad {\text{where}}\;P = \left[ {p_{1} , \ldots p_{n} } \right]$$

where

$$L_{PELM} = \frac{1}{2}\left| {\left| \beta \right|} \right|^{2} + CW\frac{1}{2}\mathop \sum \limits_{i = 1}^{n} \left| {\left| {\partial_{i} } \right|} \right|^{2} ,\;{\text{subject to:}}\;f(m_{i} )\;\beta = p_{i}^{p} - \partial_{i}^{p} , \quad i = 1 \ldots n$$

where ${\text{f}}(m_{\text{i}} )$ represents the feature mapping vector contained hidden layer with respect to $m_{\text{i}}$, and $\beta$ is the output weight vector connecting the hidden layer and the output layer. For a binary classifier, there is only one node in the output layer. Here $\partial_{\text{i}}$ represents the training error of sample $m_{i}$. It is caused by the difference of the desired output $P_{i}$ and the actual output $f(m_{i} )$ $\beta$ [28].

According to KKT theorem, the equivalent dual optimization problem with respect to (9) is

7 $$L_{{D_{ELM} }} = \frac{1}{2}\left| {\left| \beta \right|} \right|^{2} + CW\frac{1}{2}\mathop \sum \limits_{i = 1}^{n} \partial_{i}^{2} - \mathop \sum \limits_{i = 1}^{n} a_{i} (f(m_{i} ) \beta - p_{i} + \partial_{i} )$$

where ${\text{a}}_{\text{i}}$ is the constant factor of sample $m_{i}$ in the linear combination to form the final decision function, In addition, by making the partial derivatives, the Karush–Kuhn–Tucker (KKT) [30] optimality conditions are obtained [28]

8 $$\frac{{\emptyset L_{{D_{ELM} }} }}{\emptyset \beta } = 0 \to \beta = \mathop \sum \limits_{i = 1}^{n} a_{i} f\left( {x_{i} } \right)^{P} = F^{P} \varepsilon$$

9 $$\frac{{\emptyset L_{{D_{ELM} }} }}{{\emptyset \partial_{i} }} = 0 \to a_{i} = CW\partial_{i} , \quad i = 1 \ldots n$$

10 $$\frac{{\emptyset L_{{D_{ELM} }} }}{{\emptyset a_{i} }} = 0 \to f\left( {m_{i} } \right)\beta - p_{i} + \partial_{i} = CW\partial_{i} = 0, \quad i = 1 \ldots n$$

Two versions of solutions of $\upbeta$ can be derived from (10) regarding left pseudo-inverse or right pseudo-inverse. When the presented data has a small size, right pseudo-inverse is recommended because it involves the inverse of an N × N matrix. Otherwise, left pseudo-inverse is more suitable since it is much easier to compute the matrix inversion of size L × L when L is much smaller than N:

11 $${\text{When}}\;n\;{\text{is small:}}\; C = F^{P} \left( {\frac{I}{C} + WFF^{P} } \right)^{ - 1} WP$$

12 $${\text{When}}\;N\;{\text{is large:}}\; \upbeta = \left( {\frac{I}{C} + F^{P} WF} \right)^{ - 1} F^{P} WP$$

Inspired from work of [31] and the definition of a kernel [31], the output function in terms of kernel is naturally derived from the *N* × *N* version:

13 $$k\left( x \right)_{kernel} = f\left( x \right)F^{P} \left( {\frac{I}{C} + WFF^{P} } \right)^{ - 1} F^{P} WP =\,sign\left[ {\begin{array}{*{20}c} {K\left( {m,m_{1} } \right)} \\ \vdots \\ {K\left( {m,m_{n} } \right)} \\ \end{array} } \right]^{P} \left( {\frac{I}{C} + W\omega_{ELM} } \right)^{ - 1} WP$$

### Performance evaluation

In the study, we employed the following measures to evaluate the prediction performance of the Weighed-Extreme Learning Machine. The definition is shown as follows:

$${\text{Ac}} = \frac{TP + TN}{TP + FP + TN + FN}$$

$${\text{Sn}} = \frac{TP}{TP + TN}$$

$${\text{Sp}} = \frac{TN}{FP + TN}$$

$${\text{Pe}} = \frac{TP}{FP + TP}$$

$${\text{MCC}} = \frac{{\left( {TP \times TN} \right) - \left( {FP \times FN} \right)}}{{\sqrt {\left( {TP + FN} \right) \times \left( {TN + FP} \right) \times \left( {TP + FP} \right) \times \left( {TN + FN} \right)} }}$$

Ac, Sn, Sp, Pe and MCC represent Accuracy, Sensitivity, specificity, Precision and Matthews’s Correlation Coefficient, respectively. In the above formula, TP represents the number of true positives, FP is the count of false positives, TN represents the number of true negatives and FN represents the count of false negatives. In addition, for assessing the performance of the proposed classifier, we also constructed the Receiver Operating Curve (ROC) in the experiment.

## Results and discussion

### Performance of the proposed method

Using the proposed approach we performed the experiment yeast and human dataset, respectively. In order to prevent the over-fitting to affect the experimental results of the proposed approach, the experimental datasets were divided into the training datasets and independent test datasets, respectively. More specifically, 1/6 of the human datasets was selected as independent test datasets and the remaining human datasets selected as training datasets. The same strategy was also applied for the yeast dataset. In addition, for a fair comparison, we used five-fold cross-validation tests to assess the prediction performance of the proposed method in the experiment. At the same time, for ensuring fairness, several parameters of the WELM classifier were optimized in the experiment by using the grid search method. Here, we selected the ‘tribas’ function as the kernel function and set up Number of Hidden Neurons = 5000 and C = 100. The prediction results of the proposed approach on yeast and human dataset are displayed in Tables 2 and 3.

**Table 2:** Five-fold cross-validation results shown using our proposed method on *yeast*

Testing set	Ac (%)	Sn (%)	Pe (%)	MCC (%)
1	92.28	68.00	68.20	66.42
2	92.36	62.84	69.92	64.86
3	93.89	72.39	71.32	70.36
4	93.41	67.65	70.77	67.77
5	92.77	75.35	66.05	68.81
Average	92.94 ± 0.70	69.25 ± 4.80	69.25 ± 2.14	67.65 ± 0.02

**Table 3:** Five-fold cross-validation results shown using our proposed method on *human*

Testing set	Ac (%)	Sn (%)	Pe (%)	MCC (%)
1	96.83	84.03	79.08	80.42
2	96.91	81.94	81.13	80.43
3	96.72	84.95	78.64	80.58
4	96.75	84.28	79.25	80.68
5	96.58	83.28	81.91	81.60
Average	96.74 ± 0.10	83.70 ± 1.15	80.00 ± 1.43	80.74 ± 0.091

As observed from Table 2 that the proposed approach achieved good prediction results on yeast dataset, whose average accuracies of five experiments are above 92% and average Sensitivity, Precision, and MCC of 69.25, 69.25, and 67.65% respectively. Similarly, an interesting phenomenon from Table 3 is that the average Accuracy obtained is above of 96% on human dataset and average Sensitivity, Precision, and MCC of 83.70, 83.00, and 80.74% were also obtained respectively.

As displayed from Tables 2 and 3 that the proposed approach produced very good experimental results for predicting SIPs, owing to the correct choice of classifiers and feature extraction methods. A major advantage of using PSSM is to combine LAG and PCA as feature extraction methods and to employ the robust WELM classifier. This may be attributed to the following three reasons: (1) it is an obvious advantage that PSSM not only expresses the order information but also retains enough prior information. This make PSSM play a key role for improving the predicting accuracy. (2) For the sake of Local Average Group (LAG) can extract the local texture feature, the candidate self-interacting proteins can be represented by exploring the evolutionary information embedded in PSI-BLAST-constructed PSSM. This makes it possible to discover patterns of the entire sequences. In addition, for reducing the influence of noise and guaranteeing the integrity of feature vector information, the dimension of each LAG feature vector was reduced from 400 to 300 through employing PCA method. (3) The robust WELM classifier is used to calculate the classification rates. As a result, the sample information extracted by using the proposed feature extraction method is very suitable for identifying SIPs and the WELM classifier plays an important role for improving prediction accuracy.

### Comparison with the SVM-based method

It is noted that good experimental results have been obtained through using the proposed approach. However, to better assess the prediction performance of the WELM classifier, we compared the prediction performance of the WELM classifier with the SVM classifier by using the LAG feature extraction approach on yeast and human datasets. In the experiment, the LIBSVM tool [32] was employed to carry out classification. The RBF kernel parameters of the SVM were optimized by using the grid search method, where c is 0.001 and g is 0.3 and other parameters takes the default value.

**Fig. 2:**
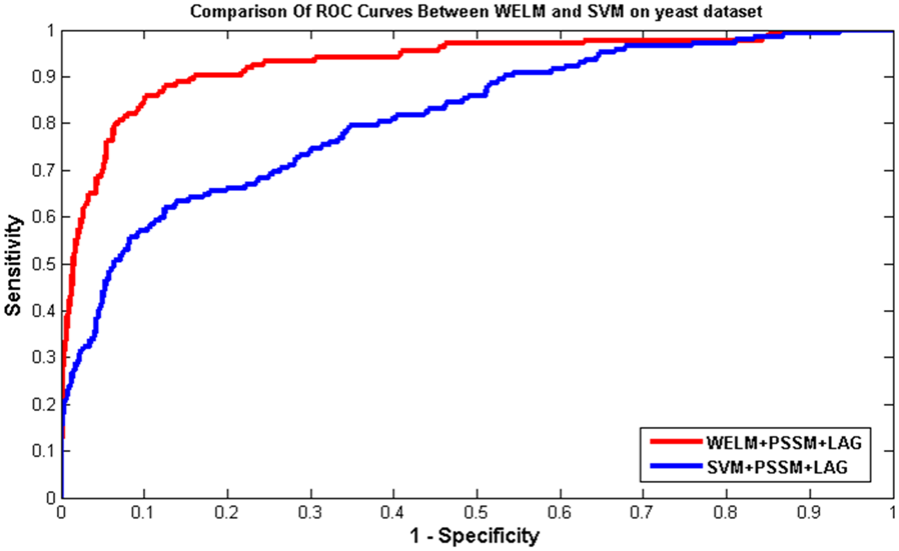
Comparison of ROC curves performed between WELM and SVM on *yeast* dataset

**Fig. 3:**
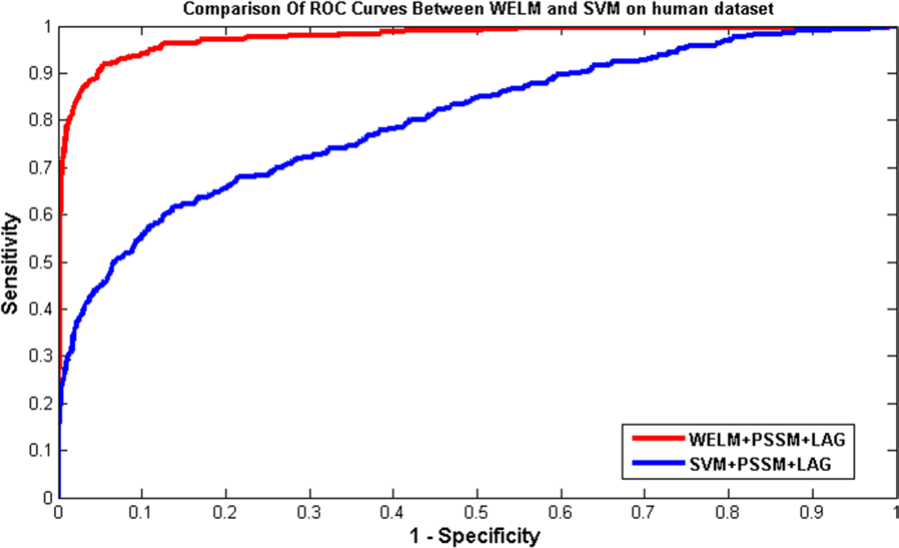
Comparison of ROC curves performed between WELM and SVM on *human* dataset

The prediction results for WELM and SVM classifiers were presented in Tables 4 and 5 on yeast and human datasets, respectively. Meanwhile, the comparison of ROC Curves between WELM and SVM were shown in Figs. 2 and 3 on yeast and human datasets, respectively. It can be seen from Table 4 that the SVM classifier gave 89.73% average Accuracy on yeast datasets. However, the WELM classifier achieved 92.94% average Accuracy. Similarly, as displayed in Table 5, 96.74% average Accuracy is obtained by the proposed WELM classifier and 93.36% average Accuracy is achieved by the SVM classifier on human dataset. These prediction results further demonstrated that the prediction performance of WELM classifier is significantly better than the SVM classifier. At the same time, it can be found from Figs. 2 and 3, the ROC curves of WELM classifier are also significantly better than the SVM classifier. This may be attributed to the reason: The WELM classifier pays attention to the samples which imply the imbalanced class distribution relative to unweight ELM. In the process of WELM classifier classification, the minority class samples are assigned with larger weight, which make the information of imbalanced class distribution is well perceived [28]. After weighting scheme is applied, WELM classifier can push the separating boundary from the minority class towards the majority class [28] As a result, the WELM classifier can be generalized to cost sensitive learning by assigning different weight [28]. In the study, the proposed prediction model obtained good prediction results. This further demonstrated that the WELM classifier is robust and effective in imbalanced data environment. Thus, all of these proved that the proposed prediction method might become useful tools for predicting SIPs, as well as other bioinformatics tasks.

**Table 4:** Five-fold cross-validation results shown by using our proposed method on *yeast*

Testing set	Ac (%)	Sn (%)	Pe (%)	MCC (%)
SVM + PSSM + LAG				
1	89.15	21.33	65.31	37.20
2	89.63	20.27	73.17	37.84
3	90.51	13.43	81.10	33.44
4	90.11	15.44	72.41	32.96
5	89.28	28.87	64.06	42.83
Average	89.73 ± 0.57	19.87 ± 6.01	71.19 ± 6.85	36.85 ± 0.04

**Table 5:** Five-fold cross-validation results shown by using our proposed method on *human*

Testing set	Ac (%)	Sn (%)	Pe (%)	MCC (%)
SVM + PSSM + LAG				
1	93.32	23.26	85.90	43.64
2	93.06	22.07	89.19	43.15
3	93.47	24.57	88.75	45.50
4	93.70	26.14	74.19	43.49
5	93.25	23.59	93.42	45.52
Average	93.36 ± 0.24	23.92 ± 1.53	93.54 ± 4.25	44.26 ± 0.012

### Comparison with other methods

In the study, in order to further verify the prediction performance of the proposed approach, the comparison of prediction performance between the proposed prediction method called WELM–LAG and three existing SIP predictor SLIPPER [1], CRS [22], SPAR [22] and three PPI predictors DXECPPI [33], PPIevo [34] and LocFuse [35] based on the human and yeast datasets are given. These comparison results were shown in Tables 6 and 7 on yeast and human datasets. It can be observed from Table 6 that the average prediction accuracy obtained by the proposed approach is obviously better than other six methods on yeast dataset. Similarity, we can find from Table 7 that the prediction accuracy of our approach is also significantly higher than six different methods on human dataset. These comparison results further proved that the proposed prediction method called as WELM–LAG is robust and efficient relative to current existing approaches. Owing to the use of a robust classifier and an effectively feature extraction method, our prediction approach obtained good prediction results. This makes the proposed method become a useful tool for predicting SIPs.

**Table 6:** Comparison of predicting performance between our method and other methods on yeast dataset

Model	Ac (%)	Sp (%)	Sn (%)	MCC
SLIPPER [1]	71.90	72.18	69.72	0.2842
DXECPPI [33]	87.46	94.93	29.44	0.2825
PPIevo [34]	66.28	87.46	60.14	0.1801
LocFuse [35]	66.66	68.10	55.49	0.1577
CRS [22]	72.69	74.37	59.58	0.2368
SPAR [22]	76.96	80.02	53.24	0.2484
Proposed method	92.94	69.25	69.25	0.6765

**Table 7:** Comparison of predicting performance between our method and other methods on human dataset

Model	Ac (%)	Sp (%)	Sn (%)	MCC
SLIPPER [1]	91.10	95.06	47.26	0.4197
DXECPPI [33]	30.90	25.83	87.08	0.0825
PPIevo [34]	78.04	25.82	87.83	0.2082
LocFuse [35]	80.66	80.50	50.83	0.2026
CRS [22]	91.54	96.72	34.17	0.3633
SPAR [22]	92.09	97.40	33.33	0.3836
Proposed method	96.74	80.00	83.70	0.8074

## Conclusion

In the work, we developed a novel computational approach termed WELM–LAG to predict SIPs by only using protein sequence information. The WELM–LAG method used a newly feature extraction method called Local Average Group (LAG) that can capture evolutionary information from PSSM and employed an effective and robust classifier called the Weighed-Extreme Learning Machine (WELM) to execute classification. The major improvement of our approach lies in adopting an effective feature extraction approach to represent candidate self-interactions proteins by exploring the evolutionary information embedded in PSI-BLAST-constructed position specific scoring matrix (PSSM); and then it also employs a reliable and robust WELM classifier to carry out classification. The proposed method was carried out on human and yeast datasets, respectively, which achieved very excellent prediction accuracies of 96.74 and 92.94%. At the same time, we also compared our method with the SVM classifier and other existing approaches on human and yeast datasets. The experimental results proved that our WELM–LAG model can extract the hidden key information beyond the sequence itself and obtain much better prediction results than previous method. It is proved that the WELM–LAG method is fit for SIPs detection and can execute incredibly well for identifying Sips. In addition, the link address (https://github.com/ajysjm/WELM_SIP_Prediction) provided the datasets and source code that can be downloaded by users. We also developed a freely available web server called WELM-LAG-SIPs to predict SIPs. The web server is available at http://219.219.62.123:8888/WELMLAG/.
